# Resistance Welding Quality Through Artificial Intelligence Techniques

**DOI:** 10.3390/s25061744

**Published:** 2025-03-12

**Authors:** Luis Alonso Domínguez-Molina, Edgar Rivas-Araiza, Juan Carlos Jauregui-Correa, Jose Luis Gonzalez-Cordoba, Jesús Carlos Pedraza-Ortega, Andras Takacs

**Affiliations:** Facultad de Ingeniería, Universidad Autónoma de Querétaro, Querétaro 76010, México; luisdomo90@gmail.com (L.A.D.-M.); erivas@uaq.mx (E.R.-A.); jc.jauregui@uaq.mx (J.C.J.-C.); jose.gonzalez.cordoba@uaq.mx (J.L.G.-C.); caryoko@yahoo.com (J.C.P.-O.)

**Keywords:** resistance spot welding, electrode force, welding current, welding time, convolutional neural network, thermal images, visible images

## Abstract

Quality assessment of the resistance spot welding process (RSW) is vital during manufacturing. Evaluating the quality without altering the joint material’s physical and mechanical properties has gained interest. This study uses a trained computer vision model to propose a cheap, non-destructive quality-evaluation methodology. The methodology connects the welding input and during-process parameters with the output visual quality information. A manual resistance spot welding machine was used to monitor and record the process input and output parameters to generate the dataset for training. The welding current, welding time, and electrode pressure data were correlated with the welding spot nugget’s quality, mechanical characteristics, and thermal and visible images. Six machine learning models were trained on visible and thermographic images to classify the weld’s quality and connect the quality characteristics (pull force and welding diameter) and the manufacturing process parameters with the visible and thermographic images of the weld. Finally, a cross-validation method validated the robustness of these models. The results indicate that the welding time and the angle between electrodes are highly influential parameters on the mechanical strength of the joint. Additionally, models using visible images of the welding spot exhibited superior performance compared to thermal images.

## 1. Introduction

Resistance spot welding (RSW) is a manufacturing process that joins two metallic materials by applying high current generating heat and pressure to the components to create the welding spot. The RSW process is known for its high precision and repeatability, ensuring consistent quality and obtaining a solid and durable joint [1,2].

This process is widely used in the automotive and aerospace industries, as well as various manufacturing applications, due to its low cycle time, low operating cost, ease of implementation, and flexibility with respect to adapting to different combinations of materials such as steel, aluminium, and titanium, among others [1]. Consequently, it is essential to ensure the quality of the joints in the resistance welding process since a malfunction can represent a risk to the end user as the assembly fails to fulfill its mechanical and functional requirements. This production error can have severe safety implications when manufacturing medical products or aerospace assemblies.

The mechanical strength evaluation obtained from the RSW process can use destructive or non-destructive testing. Destructive tests (DTs) such as pull, chisel, peeling, and tensile tests quantify the mechanical strength obtained from the joint but modify the physical properties of the components. Therefore, only a sample from an Acceptable Quality Level (AQL), not 100% of the parts [1,3], was tested. International standards such as AWS 8.9M-2012 [4], ISO 15614-12:2021 [5], and ISO 10447:2015 [6] regulate these methods. Although DT is more accurate and gives more information about the RSW quality, this process takes time, generates high costs [7,8], and cannot evaluate every welded piece.

On the other hand, non-destructive testing (NDT) is the preferred option in characterizing the welding joints since its evaluation “does not alter physical, chemical, mechanical, or dimensional properties and can be applied at any process stage” [9]. NDT offers a good solution in an automated environment to evaluate RSW quality quickly and in a cost-effective manner. However, the industry still uses human experts, which is a source of uncertainty in evaluation [10] because it is costly to integrate the methods researched in a laboratory environment into a real-life manufacturing environment [11].

Multiple studies have investigated the materials’ local mechanical properties, correlating the material response to an external force without altering the weld; for example, ref. [12] uses vibratory stress relief to evaluate dissimilar steel welding quality, ref. [13] applies residual stresses to evaluate cruciform weld joints. The work of [14] correlates the local mechanical properties of the welded material with triangulated 3D image information.

The most common approaches to reduce time loss in production associated with quality inspections are preventing the creation of defective parts (during the process) and identifying faulty parts (after the process) [11]. For precluding, the welding systems continuously monitor its parameters to identify anomalies. For identification, the experts evaluate and categorize the resulting product as acceptable or defective.

The input settings of the process define the properties of the RSW, where the parameters of electrode pressure, current, and welding time play a fundamental role. The quality of the weld is highly dependent on the accuracy and control of these parameters [15]. The welding time and current cause temperature distribution in the weld zone and melting point growth, and applying electrode pressure ensures proper contact between the welded parts [16]. It is essential to have a system that continuously monitors their evolution over time as most of these parameters vary during the melting point formation [17]. The monitoring allows the identification of any anomaly during the process.

The investigation presented in [18] implemented a real-time system based on wavelet threshold analysis to monitor and validate the temperature field within a virtual environment. They report high precision, although they took into consideration only one parameter. The work of [19] observed that the displacement of the electrode (DE) recorded with a camera can help predict the input parameters, nugget size, and strength. However, the camera has to be placed in a fixed position relative to the electrodes to observe the displacement. Ref. [20] correlated the indentation displacement with the nugget growth, expulsion, and diameter, but they did not associate the results with the mechanical strength of the piece. The research of [21] was able to cluster dynamic resistance curves (DRCs), but their results strictly depend on the electrodes’ wear status. The approach of [22] predicts the quality using machine learning models trained on information about the production containing time series data (e.g., current, voltage, or resistance) and single features (e.g., wear count or operating status information). Their work predicts with minor errors the future piece, though they cannot manage random changes, and the quality is not only influenced by the linear wearing effects.

Although online methods can offer a large amount of information to predict the weld’s quality accurately, they are costly to implement, and the latency can affect the results. Also, the published methods can only predict the quality of the upcoming pieces.

There are multiple approaches to identifying faulty pieces in the production line. Ultrasonic testing is the most widely used non-destructive testing in the automotive industry, but its accuracy depends on the user’s skills [3]. Authors of [23] proposed a machine learning model for the ultrasonic test classification in the RSW process, applying classification and regression tree (CART) and Random Forest techniques as pattern-recognition tools. They classified the welding spots into four categories: good weld, undersized weld, stick weld, and no weld. Although it is considered the most accurate evaluation method [24], it is limited to the ultrasonic beam orientation as it has to be perpendicular to the tested surface [25]. Also, it is challenging to apply restricted irregular surfaces [24], and a skilled operator is needed to interpret the signals.

Another popular non-destructive testing method is thermal imaging, which determines the quality of the RSW joint by estimating the area of the welding spot nugget. This method ensures an effective joint of the components and ensures quality.

The work of [26] used thermal images to determine the welding nugget diameter, using the color gradients for the calculation of the dimension and verifying with a visual inspection, obtaining a difference of 22% compared to the naked eye measurement of the melting point. However, the setup of their lock-in technique can be time-consuming and more expensive compared to other thermographic techniques.

To determine the quality of dissimilar dual-phase (DP) 600 and series 300 stain steel (AISI 304), ref. [27] used thermal images obtained during the RSW to train a Convolutional Neural Network (CNN). Even though they report 97.3%, they used the dataset of [28] only, classifying the weld quality.

For the RSW process evaluation, ref. [7] presented a model using CNN and a thermal imaging camera to predict the size and shape of the melting area. The model consisted of two steps: (1) reduce the noise of the videos taken by a thermographic camera, and (2) develop a 2D CNN model to detect the adequate start time of each video and the weld center for segmentation and weld size detection. They also identified that the reflection property of dissimilar weld materials is the leading cause of the variation in prediction. Although they report desirable accuracy, they only evaluate the nugget shape based on the thermal images.

Since surface defects contain features related to the joint’s mechanical performance, ref. [3] proposed image analysis based on vision and image-processing systems to determine the surface quality during welding.

While [29] established a correlation between visual information and the welding parameters, investigating the cathode anode surface on the welding discharge using high-speed cameras, the authors of [30] used the pull-off strength, nugget diameter, and fracture mode for prediction utilizing the heat trace in an image of the weld surface with a CNN algorithm. However, accurate predictions require controlling illumination, distance, and image angle. In addition, electrode contamination and misalignment affected its performance as the surface heat trace depends on heat-transfer conditions. In addition, they are only able to predict good-quality welding.

For position detection and spot welding quality, ref. [3] proposed a CNN MobileNetV3 lightweight network. Also, they introduced a data-augmentation technique for the training process, which randomly picks, organizes, and merges four into one image and evaluates all four images at once, making the network training more robust. They detect multiple nuggets accurately but can only distinguish between good and bad quality.

Finally, ref. [2] proposed a multiscale CNN model with an attention mechanism called AcmNet for the classification of solder spots into seven types: normal, copper adhesion, edge, overlap, mutilation, splash, and twist. Even though they report high accuracy, their model can categorize only the final quality but cannot suggest possible changes in the setup parameters.

To evaluate the relation between the mechanical properties, such as mechanical strength, and the variation of input parameters, ref. [15] monitored the current, voltage, electrical resistance, and electrode pressure during the exploration of the feasibility of welding two steel materials, Quenching and Partitioning (Q&P) and Transformation-Induced Plasticity (TRIP). They reported that welding current, electrode force, and welding time were key parameters influencing weld quality. However, they can estimate this through expensive adjustments to the machinery.

To estimate the influence of polarity on a resistance welding machine, ref. [28] conducted a study by recording voltage and current parameters using a Rogowski coil. Additionally, they used a thermographic camera to record the temperature. They calculated the influence of two polarities on the surface appearance, fusion diameter, and the failure modes measured with a pull-out test. Interfacial failure (IF) occurs when there is a break in the weld zone, and pull-out failure (PF) mode where the break occurs in the base material adjacent to the fusion zone. The results also show no significant correlation between the nugget diameter and the welding time.

A Random Forest model classified the weld quality into three categories (cold welds, expulsion, and good welds) in the work of [31] by capturing electrical parameters such as voltage, current, and dynamic resistance calculation. They successfully connected the process parameters with the results; however, their model’s accuracy indicates that there is still a need for a human expert during the evaluation.

For weld quality estimation, ref. [32] proposed a method utilizing the current, voltage, and dynamic resistance parameters obtained during the process. These input parameters are converted into images and then processed using a CNN. Sensors recorded the voltage, current, and clamping force during welding. Then, they calculated the dynamic resistance based on the voltage’s instantaneous values and the sensor’s sampling period during the cycle. The reported accuracy in this work is 98%, but in some cases, it was overfitting.

Similarly, ref. [33] proposed an in-line system monitoring current and voltage. The model classified the weld quality during the current signal analysis into three categories: bad welds, good welds, and metal expulsion welds. Then, these signals served as input to an artificial neural network and regression model. The presented model can predict the nugget size and quality based on current and voltage. However, it cannot predict the hidden structural characteristics.

Finally, ref. [34] monitored the current and voltage parameters to determine the mechanical resistance. They also explored the relationship between electrode pressure and dynamic resistance. According to the report, the electrode pressure affects the initial peak of the dynamic resistance. In the study, two regression models and a neural network predicted the melt diameter from the dynamic resistance, and a principal component analysis (PCA) reduced dimensionality and computational cost. However, they failed to explain the dataset characteristics they used as input information.

Moreover, the works of [33,34] use a Rogowski coil, a popular choice for measuring high-current applications with a lower limit of 1.0 kA and an upper limit of 2.4 kA. However, these coils are susceptible to external magnetic fields, which affects measurement accuracy—compared to current transformers that are less sensitive to external magnetic interference [35], making them a more reliable option in environments with high electromagnetic activity. Also, Rogowski coils can have other drawbacks in high-frequency applications, limiting their versatility and accuracy in these scenarios. They also require an integrator circuit for signal conditioning, which increases the system’s complexity.

The works mentioned above primarily focused on the force parameter assigned as input when recording electrode force. While this approach helps monitor and control the force applied in the operation, it overlooks the potential of real-time force measurements during the operation. Furthermore, the examined computer vision articles utilize CNN to evaluate and categorize the weld quality using only the visual outcome of the nugget. These approaches lose the opportunity to connect the input variables of the RSW process with the result images due to the complex nature of the experiment setup.

This study proposes a non-destructive RSW evaluation framework using computer vision and machine learning models to connect the welding spot’s mechanical characteristics and process parameters with the nugget’s image information. The trained models have practical applications in welding and manufacturing, as the automated process requires cheap and continuous quality monitoring and feedback systems. The proposed framework can train models that not only classify the quality of the welded nugget but also provide valuable insights into the welding process parameters using only image information. A spot welding test bench was assembled and used for the dataset generation, where the system continuously monitors the applied force and current over the welding spot’s formation. This work proposes six machine-learning models to correlate thermal and visible images with the process parameters. These models use CNNs to classify the fusion quality and predict the welding spot’s mechanical characteristics and process parameters.

## 2. Materials and Methods

The methodology applied in the present work consists of four steps: (1) use of a manual resistance spot welding machine to study the impact of the input parameters vs. quality attributes; (2) creation of a dataset for the training of the artificial intelligence models; (3) evaluation of the quality of the weld; and (4) testing the effectiveness of the model to detect defects in the welded joints.

### 2.1. Use of Resistance Spot Welding Machine

One of the key factors influencing the quality of the resistance welding process is the weld spot’s formation. This formation is directly influenced by three essential parameters: electrode force, welding current, and welding time. These are considered the main parameters of resistance spot welding, and each plays an essential role in forming a quality weld [36].

Electrode pressure: The amount of pressure the electrodes applied to the metal sheets during the welding process [37]. It must be strong enough to overcome material distortion for optimum contact before current flows but not too strong to avoid problems. This process affects the quality and strength of the weld and is commonly quantified in newtons (N).

Welding current: The amount of electric current used during welding [37] affects the heat generated during welding and is generally measured in amperes (A). Keeping the welding current as low as possible in each application is advisable. A too-high current might expel molten material from the weld nugget and stick to the tip of the electrodes. The current in the resistance welding process is in the range of thousands of amperes [2]. One method to determine an acceptable range of current is using the Lobe curves of the welding [38] against different factors such as material type, component thickness, and applied cycle time.

Welding time: The electric current flowing through the electrodes to the metals generates the melting point to create the welding spot [37]. This parameter is typically measured in milliseconds (ms). Additionally, the compression time is used to stabilize the force applied to the parts, and the waiting time after welding is necessary for the weld to solidify before releasing the parts.

**Figure 1:**
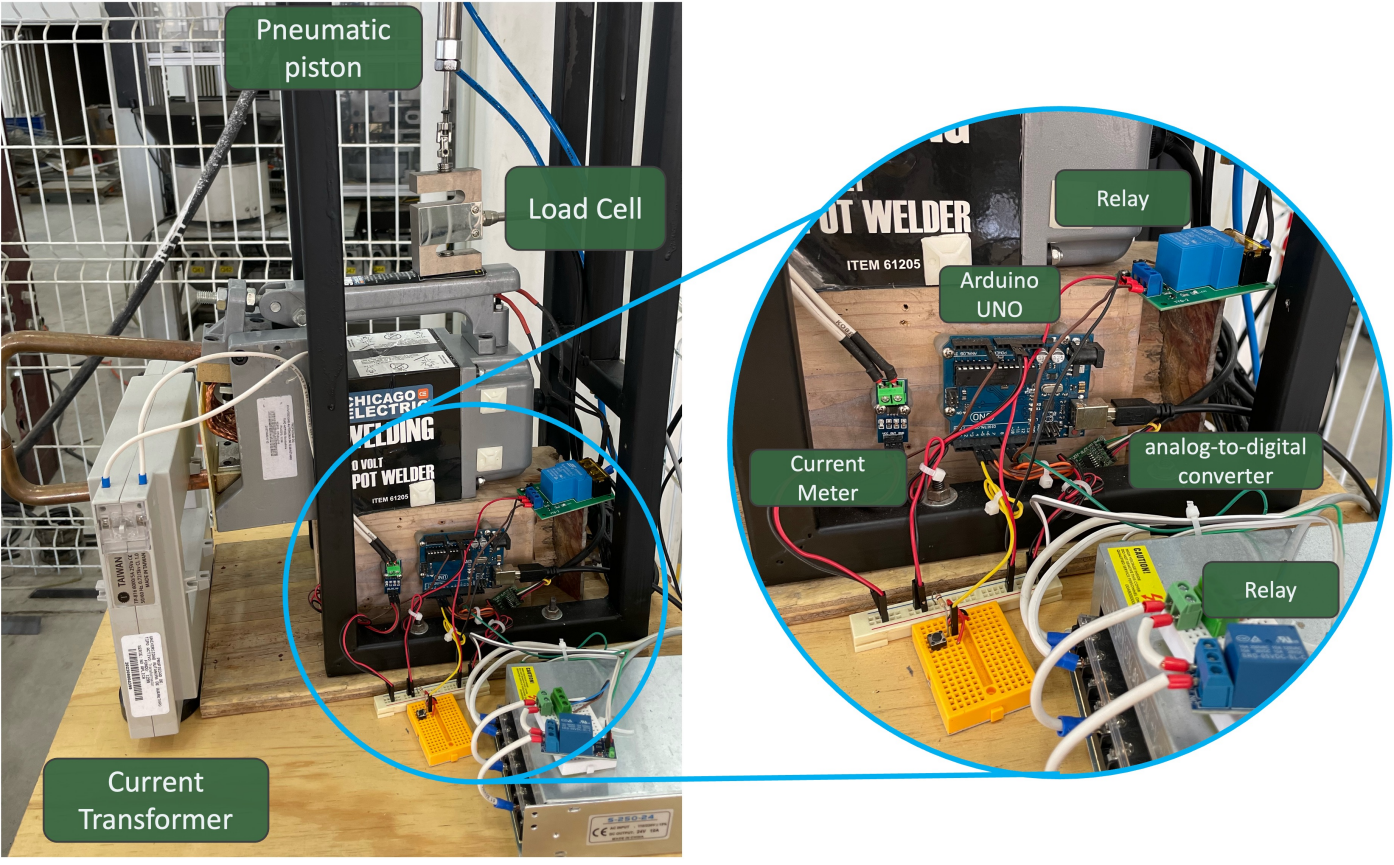
Use of RSW machine.

The resistance welding machine Chicago Electric model 61205 was used [39] (Figure 1) for the experiment consisting of the following units:

- A load cell to record the applied force to the electrodes with a frequency of 10 samples per second (SPS) using a 24-bit analog-to-digital converter for high sensitivity.
- A current transformer that connects to a current meter to provide reliable measurements.
- A current meter to measure the current with a frequency of 10 SPS.
- An electronic embedded system called Arduino UNO to control the system, setting the force application process’s cycle time, compression time, and dwell time parameters.
- A pressure gauge with a pneumatic cylinder and a solenoid valve to regulate the electrodes’ applied force.

### 2.2. Dataset Generation

#### 2.2.1. Sample Generation

The construction of the samples was a result of a systematic approach, carefully considering the combinations of the weld time, the angle between electrodes, and electrode pressure setup parameters. The weld time, for instance, varied in a range of [400–1200 ms], with increments of 200 ms, ensuring a comprehensive exploration of the parameter space. Two settings of electrode tips were used to vary the angle between electrodes, each chosen with a specific purpose in mind. The first one had a 0-degree angle (parallel contact surfaces), while the other had a 15-degree angle between the contact surfaces and the electrodes. Two pressure ranges of 60 and 80 PSI were defined for sample construction, and a decision was made after careful consideration of the requirements.

The samples were in strict accordance with the industry standard AWS 8.9M-2012 [4] during construction using AISI 1010 [40] steel plates (material characteristics in Table 1).

**Figure 2:**
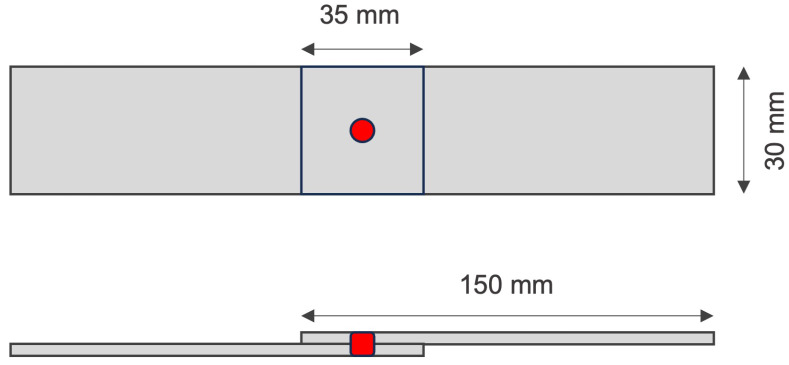
Size and configuration of weld samples.

The plates were made to overlap and were welded together, adhering to the highest standards. The geometric dimensions of the test specimens are presented in Figure 2.

A total of 495 samples—falling into three categories: bad, good, and expulsion—were built with the combinations of the established parameter range in a complete factorial design of experiments (Table 2). Among them, 32 samples were built with a random combination of the parameters to avoid data bias and to evaluate the influence of the input parameters of welding time, electrode pressure, and angle between the electrodes with the mechanical maximum force. To create “bad” samples, a combination of low and high setups of the cycle time and 15º angle between electrodes were set. For the “good” examples, the central parameters of the cycle time and a 0º angle between electrodes were used. Finally, the “expulsion” samples were created with the parameters of long welding time and 15º angle between electrodes. Each sample was labeled with an identification number to maintain the traceability of the used parameters for its production to integrate the information into the dataset.

Before starting the resistance welding procedure, the system registered the component characteristics, including material type and thickness, and process input parameters, such as pneumatic cylinder pressure, cycle time, and electrode angle. During welding, the system continuously recorded the electrode force and current parameters. At the end of the process, visible images from both sides of the welded plates and a thermal image of the melting point were captured. In addition, the maximum pull force and the welding nugget diameter were also documented.

The framework used two different types of images: thermal and visible images. Visible images are captured by sensors detecting visible light wavelengths between 400 nm and 700 nm. The detected information is digitalized in three channels (red, green, and blue). The thermal images are single-channel imaging using false colors to represent the views detected in the long wave infrared band ranging between 7.5 μm and 14 μm.

The visible images used a 15 cm distance between the camera and the sample surface, which was approximately 10 cm in the case of the thermal images (captured by a Fluke Ti105 infrared camera). As it is difficult to record the heat information during welding due to metal reflection, thermal images were captured immediately after (∼one second) during the cooling phase. Nevertheless, the images show the heat-affected zone’s size, shape, and propagation.

**Figure 3:**
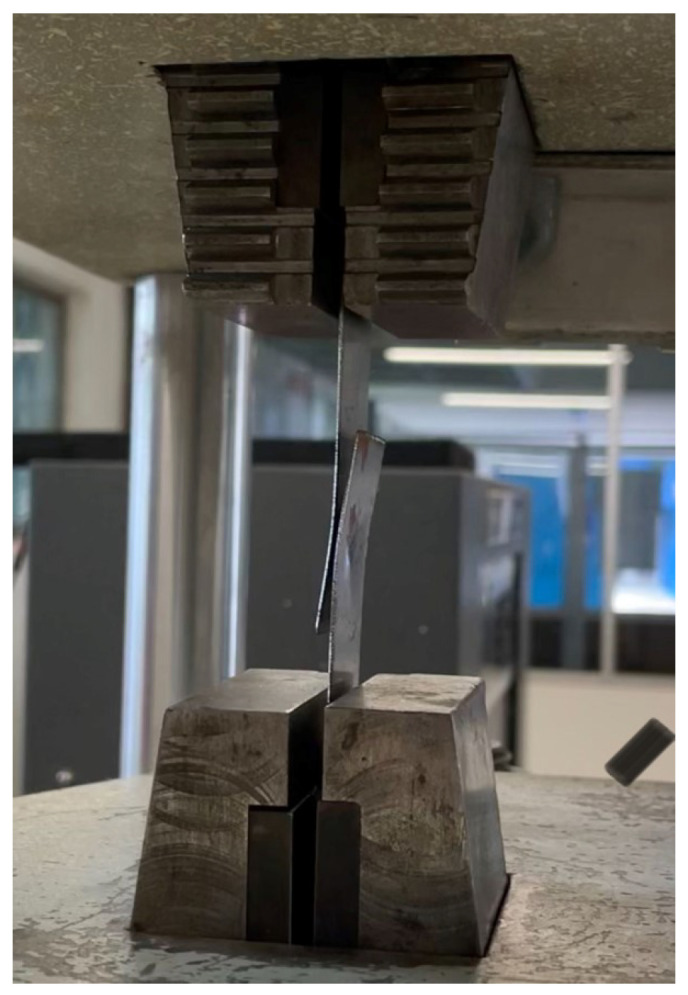
Pull force experimental setup with Tinus Olsen Testing machine.

Tensile tests were performed with a Tinus Olsen testing machine with a maximum load capacity of 300 KN and a resolution of 0.1 N. For the pull test, the metal plates were aligned between two V-shaped jaws with a knurling that prevented the parts from being displaced during the test (Figure 3). Subsequently, the maximum pull force value was recorded when the welded parts reached the breaking point due to the applied force.

The speed used for the testing was 2 mm/min. The positioning of the parts was measured in order to ensure the accurate alignment and centering of the specimens with the jaws of the testing machine prior to testing.

**Figure 4:**
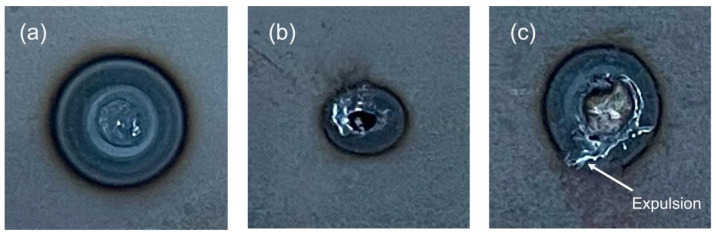
Weld spot classification: (**a**) good, (**b**) bad, (**c**) expulsion.

After testing, the nugget diameter was measured with an electronic caliper with a resolution of 0.01 mm and categorized into good, bad, and expulsion samples (Figure 4). The “expulsion” classification was selected because it impacts the functional performance of the fusion since part of the material is splashed out of the welding area, weakening the joint.

#### 2.2.2. Image Processing

Because the spot weld regions only occupy a small portion of the total area, digital and thermal images were processed to segment the weld region and resize the image. Additionally, noise and unwanted features were removed to improve the image quality.

During the image pre-processing, to acquire pixel-level information, Geeqie software [42] was used to locate the center point of the weld. Coordinates of the welding spot center in each image were manually recorded in a CSV file. Subsequently, using Python programming language with the OpenCV library [43], the images were cropped to a size of 350 × 350 pixels using the center point ± 175 pixels on the x-axis and center point ± 175 on the y-axis for the visible images. The algorithm then cropped thermal images to 300 × 300 pixels and incorporated a 10 mm scale line as a welding nugget measurement reference.

Spatial resolution for thermal images of 0.0625 mm/pixel and 0.06535948 mm/pixel for surface images.

**Figure 5:**
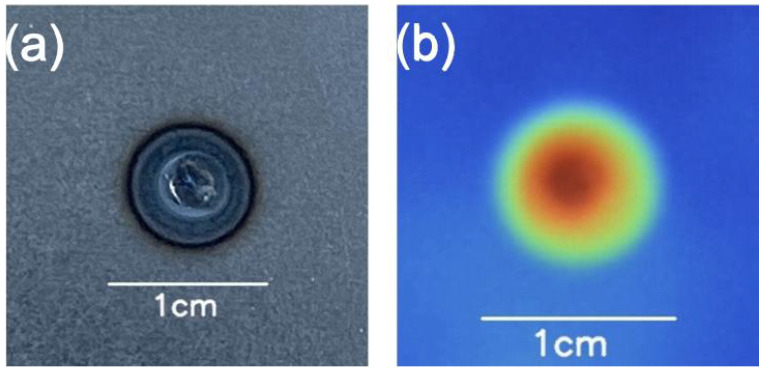
Example of (**a**) visible image of the spot welding process standardized to 350 × 350 pixels and (**b**) thermal image of the spot welding process standardized to 300 × 300 pixels.

Visible images (Figure 5a) of each sample were taken from the front (F) and back (B) of the spot. Thermal images (Figure 5b) were named according to the sample identification number.

#### 2.2.3. Data Assembly

The dataset—published in the Mendeley repository [44] and described in [45]—was generated with a total of 495 units, constructed with different welding parameters by varying electrode pressure, angle between the electrode, and welding times. The setup and the recording parameters were saved in a comma-separated values (CSV) file.

The dataset’s (Table 3) first three rows after the ID number (attributes 2, 3, and 4) contain the initial input parameters: the initial pressure value supplying the pneumatic circuit; the welding time; and the angle between the contact surfaces and the electrodes.

The second section includes the recording attributes (attributes 5 and 6), the electrode pressure, and the welding current. The sample rate of these measurements was ten samples per second during the welding. However, due to the diversity of welding times, samples have different data lengths for these parameters. Therefore, it was necessary to use measures of dispersion of the data as the minimum, maximum, and average value of each piece to be used in the Convolutional Neural Network.

The third section (attributes 7 and 8) contains features that describe the characteristics of the material: the material thickness, the recorded thickness of both metal plates in micrometers, and the type of material, which was only AISI 1010 quality carbon steel.

The fourth section (attributes 9 and 10) contains information about the weld quality, including the recorded fusion diameter after performing the pull test with an electronic vernier and the maximum force value obtained during the pull test.

Finally, the visual attribute of quality classification (attribute 11) is defined by three classes: good, bad, and expulsion (class balance in Table 4, and range of attributes in Table 5).

In addition, the dispersion of the data (Table 5).

### 2.3. Machine Learning Model Creation

#### 2.3.1. Convolutional Neural Network Development

As the dataset is unique, different state-of-the-art Convolutional Neural Networks were built based on four state-of-the-art architectures to choose the best-performing model. MobileNet, ResNet50, and DenseNet201 were selected as high-performing RSW evaluation models based on [2] evaluation and comparison. The LeNet-5 model was selected for its simplicity and speed, and according to [46] with similar accuracy as the previous models. The networks were trained and evaluated to extract features in thermal and non-thermal images related to changes in process parameters.

After selecting the architecture, six models were built to evaluate the quality of the resistance welding process and indicate the input parameters. Three models were trained on visible images, while the others used thermal images.

Each model has its own input and output characteristics:

1. Quality by visible images: This is a quality classifier model based on visible images. The output categories are “good”, “bad”, and “expulsion”.
2. Quality by thermal images: This is a quality classifier model based on thermal images. The output categories are “good”, “bad”, and “expulsion”.
3. Value prediction by visible images: This is a model that predicts the quantitative parameters of the nugget diameter and pull strength, based on images of the melting point surface.
4. Value prediction by thermal images: This is a model that predicts the quantitative parameters of the nugget diameter and pull strength, based on thermal images of the melting point.
5. Input parameter prediction by visible images: This is a model that indicates the input parameters of pneumatic cylinder pressure, electrode angle, material thickness, welding time, electrode force (minimum and maximum), and maximum welding current, based on visible images.
6. Input parameter prediction by thermal images: This is a model that indicates the input parameters of pneumatic cylinder pressure, electrode angle, material thickness, welding time, electrode force (minimum and maximum), and maximum welding current, based on thermal images.

Among the four tested state-of-the-art architectures (MobileNet, LeNet-5, ResNet50, and DenseNet201), the LeNet-5 model was the best for its simplicity and low computational cost. The model architecture was set for a 350 × 350 input array for visible images with three output neurons for the different classes.

Architectures without final layers were imported for the other models, and new ones were added to reconnect with the model and process the information. “Flatten” layers were added to flatten the data: a dense layer with sigmoid activation of 84 neurons and, finally, a dense layer with softmax activation of 3 output neurons.

All models were initially trained with the hyperparameters (Table 6) as in [2] for comparison.

#### 2.3.2. Training of the Models

**Figure 6:**
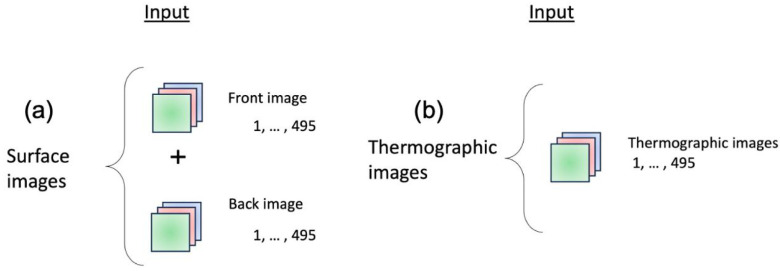
Input array for (**a**) visible image-based models and (**b**) thermal image-based models.

An array was generated to store the input images of the six models and to be used as input for the different models based on visible images (Figure 6a) or thermal images (Figure 6b).

For the output labels of models 1 and 2, an array in “OneHot” format was generated: [1. 0. 0. 0.] “bad”, [0. 1. 0. 0.] “good”, [0. 0. 1. 0.] “expulsion”. In models 3 and 4, an array with the values of the pull-off force and nugget diameter was created for the quality parameter outputs. Finally, an output array was generated for models 5 and 6, which contains the input welding parameters pneumatic cylinder pressure, electrode angle, material thickness, welding time, electrode pressure (minimum and maximum), and maximum welding current.

These arrays were randomly split into training (60%), validation (20%), and test (20%) groups according to the 60-20-20 rule. Finally, the input data were normalized according to each model’s processing.

The training results of each model were compared between the selected architectures to select the best model. Since models 1 and 2 are classification networks, they used accuracy for the evaluation. The others are prediction models, which use mean absolute error (MAE) as a metric.

#### 2.3.3. Hyperparameter Search

Once the best architecture was defined for each model, a search for hyperparameter optimization was performed using Keras Tuner [47]. This framework can search with Bayesian optimization, hyperband, and random search algorithms. For this study, a random search was performed in the search space described in Table 7.

### 2.4. Analysis of Results

To ensure the measurement’s accuracy and reliability, the parameters of force applied to the electrodes and current were calibrated, and multiple tests with different ranges were performed to determine the measurement’s repeatability.

For the load cell, multiple constant masses of 266 g were used, previously weighed with a calibrated scale. The number of masses was changed to increase the weight from 0 g to 2128 g, and 31 measurement points were taken for each step.

In the current measurement, multiple voltages were applied using an AC voltage regulator and a constant resistor to generate different current ranges, which were verified using a Fluke 177 multimeter, which has a resolution of 0.01 mA and can measure a maximum current of 10 A. Tests were conducted within a current range of 0 A to 5 A, with increments of 0.5 A, taking a total of 35 data points at each step.

Finally, the force applied to the circuits was measured by adjusting the supply pressure in different ranges to determine the relationship between the supply pressure and the measured force. The process involved manually activating the solenoid valve and recording 121 data points per sample. The range of applied pressure was from a minimum pressure of 35 PSI (necessary for valve activation) to a maximum of 95 PSI (maximum pressure supported by the pneumatic cylinder). Thirty samples were recorded at each step to ensure repeatability.

After the workbench was tested, the framework evaluated two areas: 1. how much the input parameters affect the output; 2. how robust the machine learning models are.

#### 2.4.1. Parameter Correlation

A complete factorial experimental design was developed with four repetitions of each combination for 32 samples to evaluate the effect of the RSW process input parameters (welding time, angle between electrodes, and pressure in the pneumatic cylinder) against the mechanical strength.

A Pearson correlation matrix [48] was used to determine the relationship between the input and quality parameters. The welding current and electrode force parameters were used as dispersion measures of the time series’ minimum, maximum, and average value data.

Pearson correlation (Equation (1)) is a statistical measure that evaluates the linear relationship between two continuous variables. It determines the degree of association, with 1 being a positive association between two variables, −1 a negative association between said variables, and a value close to 0 if the variables do not present a linear relationship between them [48].

(1) $$r=\frac{\sum_{i=1}^{n}\left(x_{i}-\bar{x}\right)\left(y_{i}-\bar{y}\right)}{\sqrt{\sum_{i=1}^{n}{\left(x_{i}-\bar{x}\right)}^{2}\sum_{i=1}^{n}{\left(y_{i}-\bar{y}\right)}^{2}}}$$

where:

- *r* —Pearson’s correlation coefficient;
- *n*—Number of data points;
- $x_{i},y_{i}$—Individual data points of variables *x* and *y*;
- $\bar{x},\bar{y}$—Means of *x* and *y*, respectively.

#### 2.4.2. Model Performance

The effectiveness of each model was verified using the cross-validation strategy using groups of k = 4, 5, and 9. The classification models calculate the confusion matrix, where the True Positive (TP) represents the number of points where the class prediction equals the actual value, and the True Negative (TN) shows correctly categorized non-class points. The False Positive (FP) is where a non-class classification is expected, and the class is predicted, and the False Negative (FN) is where the model classifies a non-class point as a class. The framework calculated accuracy (Equation (2)) to compare the performance with other works in the state of the art, and the F_1_ score [49] (Equation (3)) was used to overcome accuracy’s limitations and to have a more accurate evaluation of the classification power of the models.

(2) $$\mathrm{Accuracy}=\frac{\mathrm{TP}+\mathrm{TN}}{\mathrm{TP}+\mathrm{TN}+\mathrm{FP}+\mathrm{FN}}$$

(3) $$F_{1}=2*\frac{\mathrm{Precision}*\mathrm{Recall}}{\mathrm{Precision}+\mathrm{Recall}}$$

where:

$$\begin{array}{cc} \mathrm{Precision} & =\frac{\mathrm{TP}}{\mathrm{TP}+\mathrm{FP}}\\ \mathrm{Recall} & =\frac{\mathrm{TP}}{\mathrm{TP}+\mathrm{FN}} \end{array}$$

Meanwhile, the regression models used Mean Absolute Error (MAE) (Equation (4)), Mean Squared Error (MSE) (Equation (5)), Root Mean Squared Error (RMSE) (Equation (6)), Relative Absolute Error (RAE) (Equation (7)), Relative Squared Error (RSE) (Equation (8)), Coefficient of Determination (R^2^) (Equation (9)), and Correlation Coefficient (CC) (Equation (10)) metrics for evaluation, where $y_{i}$ is the real data, $\bar{y}$ is the mean of the real data, ${\hat{y}}_{i}$ is the predicted value, and $\bar{\hat{y}}$ is the mean of the predicted values.

(4) $$\mathrm{MAE}=\frac{\sum_{i=1}^{n}\left|y_{i}-{\hat{y}}_{i}\right|}{n}$$

(5) $$\mathrm{MSE}=\frac{\sum_{i=1}^{n}{\left(y_{i}-{\hat{y}}_{i}\right)}^{2}}{n}$$

(6) $$\mathrm{RMSE}=\sqrt{\frac{\sum_{i=1}^{n}{\left(y_{i}-{\hat{y}}_{i}\right)}^{2}}{n}}$$

(7) $$\mathrm{RAE}=\frac{\sum_{i=1}^{n}\left|y_{i}-{\hat{y}}_{i}\right|}{\sum_{i=1}^{n}\left|y_{i}-\bar{y}\right|}$$

(8) $$\mathrm{RSE}=\frac{\sum_{i=1}^{n}{\left(y_{i}-{\hat{y}}_{i}\right)}^{2}}{\sum_{i=1}^{n}{\left(y_{i}-\bar{y}\right)}^{2}}$$

The Coefficient of Determination [50] (Equation (9)) is a statistical measurement that shows how well the model explains the variance in the data. The value ranges between zero and one, with no explanation when it is zero, and one when the model explains all the variability.

(9) $$R^{2}=1-\mathrm{RSE}$$

The Correlation Coefficient [51] (Equation (10)) calculates the correlation between actual and predicted values. It varies between zero and one, where zero is interpreted as no correlation and one as a perfect correlation.

(10) $$\mathrm{CC}=\frac{S_{PA}}{\sqrt{S_{P}*S_{A}}}$$

where:

$$\begin{array}{cc} S_{PA} & =\frac{\sum_{i=1}^{n}\left({\hat{y}}_{i}-\bar{\hat{y}}\right)\left(y_{i}-\bar{y}\right)}{n-1}\\ S_{P} & =\frac{\sum_{i=1}^{n}{\left({\hat{y}}_{i}-\bar{\hat{y}}\right)}^{2}}{n-1}\\ S_{A} & =\frac{\sum_{i=1}^{n}{\left(y_{i}-\bar{y}\right)}^{2}}{n-1} \end{array}$$

### 2.5. Computing Specifications and Libraries

This project was developed on a macbook-pro 2021, with a RAM memory of 32 GB, and an Apple M1 Max chip with a 10-nucleus CPU, 24-nucleus GPU and 16-nucleus neural engine.

The described models were developed in Python version 3.9.13 with the following libraries.

- Keras version 2.10.0;
- Tensorflow version 2.10.0;
- Numpy version 1.23.2;
- Pandas version 1.5.1;
- Matplotlib version 3.7.3;
- Seaborn version 0.12.2;
- Opencv version 4.6.0.66.

## 3. Results

### 3.1. Test Bench Evaluation

The results of the test bench evaluation were consistent with the load cell, circuits, and pressure level. The test bench can be trusted to measure the applied force as the accuracy of the load cell for a wide range of weight measurements was in the correct range (Table 8).

Also, the accuracy of the circuit and its measurements demonstrate consistency and repeatability (Table 9).

During the force experiment, the force was recorded in the range of 35 PSI–95 PSI, with increments of 15 PSI between tests. It has been observed that the maximum point has little variability between the 30 samples, indicating that the process is constant and repeatable.

### 3.2. Parameter Correlation

**Figure 7:**
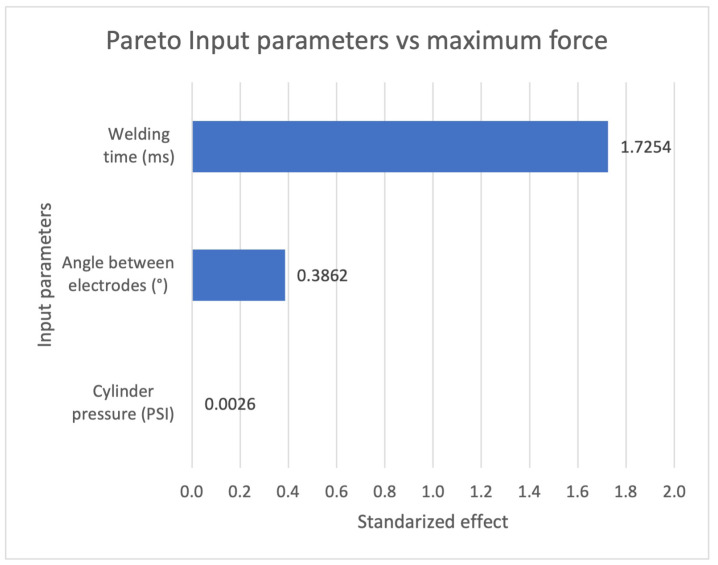
DOE Pareto effect of input parameters vs. maximum pull force.

A complete factorial design of experiments was developed with four replicates of each combination for a total of 32 samples to evaluate the effect of the input parameters of the RSW process (welding time, angle between electrodes, and pneumatic cylinder pressure). Their effect on the process parameters vs. mechanical strength was evaluated (Figure 7), indicating that the welding time factor is the most significant, followed by the angle between electrodes, and finally, the pneumatic cylinder pressure, which did not affect the pull-out force

**Figure 8:**
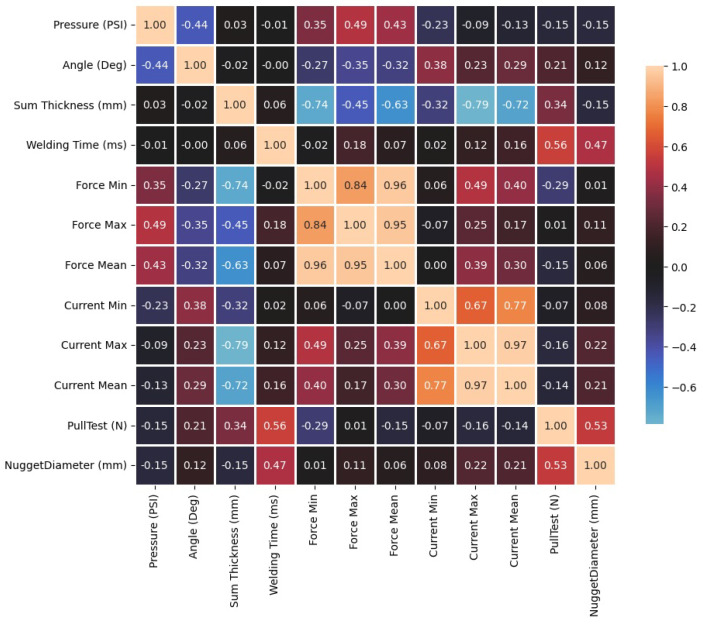
Pearson correlation matrix.

A Pearson correlation matrix was performed (Figure 8) to determine the relationship between the input and quality parameters. The welding current and electrode force were represented in the calculation with their minimum, maximum, and average value data of the time series.

The Pearson correlation matrix indicates that among the input parameters, the welding time has the highest correlation with the quality parameters of pull-off force and nugget diameter of 0.55598 and 0.472734. From the electrode pressure data, the minimum and maximum force correlated the most with the quality parameters of −0.28668 and 0.114633. On the other hand, in the welding current time series, the maximum current is the most correlating parameter with −0.164266 and 0.219147.

### 3.3. Machine Learning Model Selection

Several state-of-the-art machine learning models were tested for the project. The results of the evaluation in the case of all six models show that they all performed equally well with high validation accuracy (Table 10) in the classification models and with low mean average error in the case of the prediction models (Table 11 and Table 12). The LeNet-5 architecture came out victorious as it had the lowest computational time. After the model selection, Keras Tuner optimized each architecture with hyperparameter exploration.

### 3.4. Model Hyperparameter Tuning and Results

After the model selection, a random search of ten runs for all six models with two repetitions performed in the search space (Table 7) looking for the optimization of the accuracy of model 1 (Table 13) and model 2 (Table 14), the MAE of model 3 (Table 15), model 4 (Table 16) model 5 (Table 17), and model 6 (Table 18) in the validation.

After identifying the best model hyperparameters, k-fold cross-validation was performed using 4, 5, and 9 groups to calculate accuracy, precision, recall, and F_1_ score evaluation metrics for models 1 and 2 (Table 19) and MSE, RMSE, MAE, RAE, RSE, CC, and $R^{2}$ for models 3, 4, 5, and 6 (Table 20).

**Figure 9:**
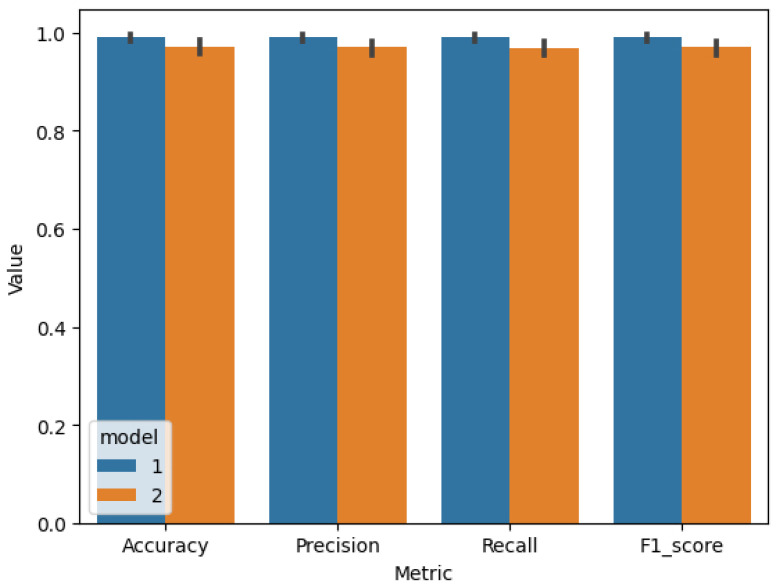
Results for cross-validation model 1 (test 6) vs model 2 (test 3).

**Figure 10:**
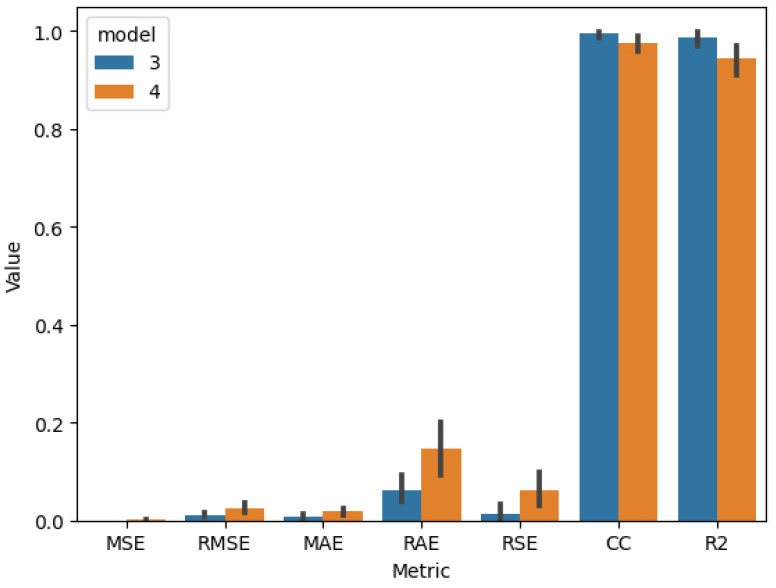
Results for cross-validation model 3 (test 2) vs model 4 (test 4).

**Figure 11:**
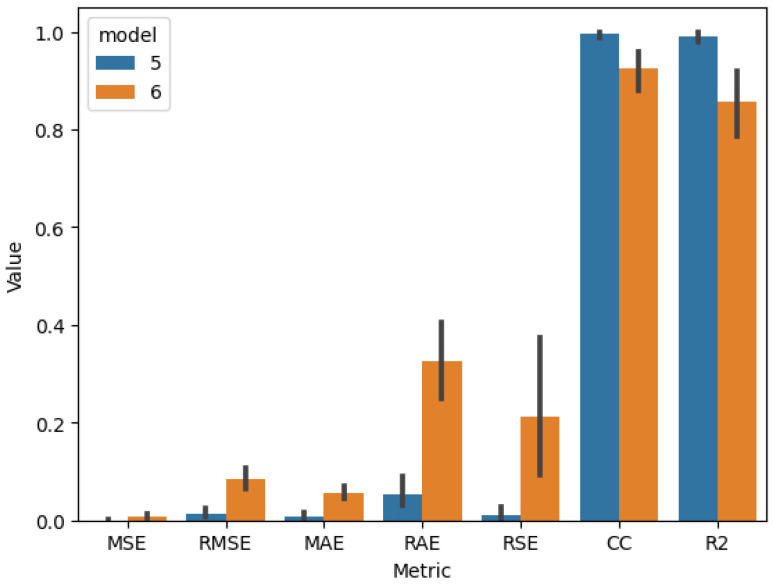
Results for cross-validation model 5 (test 3) vs model 6 (test 10).

The comparison of the cross-validation results of the visual quality-classification models (Figure 9), the quality-prediction models, and input parameter-prediction models (Figure 10 and Figure 11) proves that models based on visible images have better performance in all metrics, as well as lower variance. That indicates that these models are more robust using different training and validation sets than those using thermal images.

### 3.5. Evaluations of Example Models Predictions

**Figure 12:**
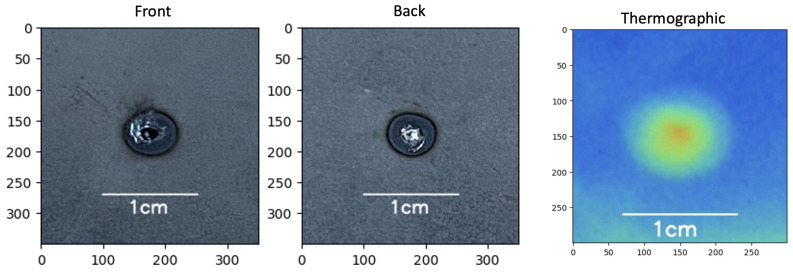
Part 480, bad sample, visible and thermal images.

**Figure 13:**
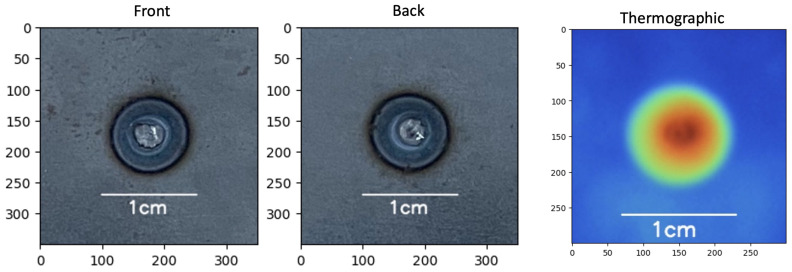
Part 474, good sample, visible and thermal images.

**Figure 14:**
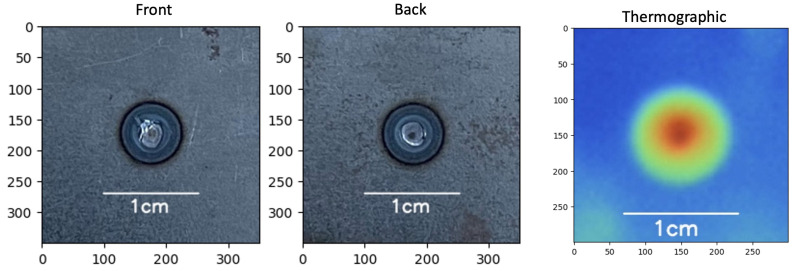
Part 385, expulsion sample, visible and thermal images.

The confidence level was calculated to measure the first two models’ robustness by evaluating them with single example images with different quality classifications (“bad” (Figure 12), “good” (Figure 13), and “expulsion” (Figure 14)).

The classification comparison of models 1 and 2 (Table 21) exhibits the strength of the model trained with visible images performing in all three classes with almost perfect confidence. In the meantime, the model trained on the thermal images only has confidence in classifying the “good” class.

The measured value was compared to the quality parameter prediction to evaluate models 3 and 4. In this evaluation (Table 22), both models predicted the pull-out force and welding nugget diameter with high accuracy and low error rate. However, the model trained with visible images performed slightly better.

As in the previous evaluation, the actual values were compared with the input parameter predictions to evaluate models 5 and 6. This evaluation (Table 23) clearly shows the superiority of the visible image trained model by predicting the input parameters (angle between electrodes, pressure in the pneumatic cylinder, thickness, welding time, minimum force recorded at the electrodes, maximum force recorded at the electrodes, and maximum current) with a much lower error rate than the thermal image trained counterpart.

## 4. Discussion

The presented comprehensive study shows a couple of significant findings that can be applied in future works and experiments.

The correlation of the input parameters shows a higher relationship between the welding time and the quality parameters of pull force and welding diameter, followed by the maximum current during the process and the angle between electrodes. In contrast, the pressure in the pneumatic cylinder does not present a correlation with the quality parameters of the RSW. In other words, machine learning models trained on simple image information cannot predict the pressure used during the welding process.

The extensive development and evaluation of machine learning models for this experiment show that the state-of-the-art LeNet-5 model emerges as the superior choice among the tested architectures. It offers a faster prediction time and demonstrates equal or superior evaluation metrics compared to the other architectures in all six models defined in this work.

Also, the models based on visible images perform better and are more robust than those based on thermal images. They have a higher average performance in cross-validation and a lower standard deviation, which indicates that these models have constant repeatability in different test groups.

## 5. Conclusions

This work shows multiple achievements during the generation of this non-destructive weld quality-prediction methodology.

A dataset integrating construction parameters, electrode current and strength monitoring, material characteristics, and melting point-related quality attributes was created. This dataset was used to train different models for classifying and predicting resistance welding quality attributes.

The developed models in the present study demonstrate a correlation between input parameters and weld spot quality in their visual and functional attributes.

Utilizing visual information, we can predict quality attributes such as category, pull force, welding diameter, and construction parameters of the spot welding process with impressive accuracy.

We can conclude that the models that use visible images as input are more accurate than those that use thermal images. That is due to the information the medium contained. In the meantime, the visible image shows the shape and form of the exploded material, and our handheld camera’s thermal imagery only captures the shape of the weld spot with the heat propagation on the surface.

Looking ahead, there is significant potential for further research. Multiple areas can be improved to enhance the quality of the environment, such as using grayscale filters and contours for image processing, expanding defect classes, and implementing different classification and prediction models in a production environment using an embedded system.

## Figures and Tables

**Table 1:** Properties of AISI 1010 carbon steel (UNS G10100) [41].

Chemical Composition	Content (%)	
Iron, Fe	99.18–99.62%	
Manganese, Mn	0.30–0.60%	
Sulfur, S	≤0.050%	
Phosphorous, P	≤0.040%	
Carbon, C	0.080–0.13%	
**Physical Composition**	**Metric**	**Imperial**
Density	7.87 g/cm^3^	0.284 lb/in^3^
**Mechanical Properties**	**Metric**	**Imperial**
Tensile strength	365 MPa	52,900 psi
Yield strength (depending on temper)	305 MPa	44,200 psi
Elastic modulus	190–210 Gpa	27,557–30,458 ksi
Bulk modulus (typical for steel)	140 GPa	20,300 ksi
Shear modulus (typical for steel)	80.0 GPa	11,600 ksi
Poisson’s ratio	0.27–0.30	0.27–0.30
Elongation at break (in 50 mm)	20%	20%
Reduction of area	40%	40%
Hardness, Brinell	105	105
Hardness, Knoop (converted from Brinell hardness)	123	123
Hardness, Rockwell B (converted from Brinell hardness)	60	60
Hardness, Vickers (converted from Brinell hardness)	108	108

**Table 2:** Experiment design parameters and ranges.

Parameter	Description	Data Type	Range
Welding time	Cycle time (ms)	Continuous	200–1500 ms
Pressure	Pressure in the pneumatic cylinder (PSI)	Continuous	35–95 PSI
Angle	Angle between electrodes (°)	Continuous	0–15°

**Table 3:** Description of data and attributes.

N.	Attribute	Format	Description
1	Sample ID	Numeric	Unique identification number for each sample
2	Pressure	Numeric	Pressure on pneumatic cylinder
3	Welding time	Numeric	Welding process time
4	Electrode angle	Numeric	Angle between the electrodes
5	Electrode force	Numeric	Force applied to the electrodes
6	Welding current	Numeric	Current passing through the metal sheet
7	Material thickness	Numeric	Thickness of the material
8	Material type	Categorical	Material composition
9	Pull test force	Numeric	Mechanical resistance of the welding join
10	Nugget diameter	Numeric	Diameter of welding nugget
11	Category	Categorical	Category of welding spot (good, bad, expulsion)

**Table 4:** Class balance.

Class	Category	Quantity	Percentage
0	Bad	31	6%
1	Good	443	89%
2	Expulsion	21	4%

**Table 5:** Dispersion of data.

Attribute	Minimum	Maximum
Pressure (PSI)	35	95
Welding time (ms)	200	1500
Angle between electrodes (°)	0	15
Electrode pressure (PSI)	0	133.53
Welding current (A)	639.81	5009.43
Thickness (mm)	0.61	1.057
Pull force (N)	1410.3	5806.5
Welding nugget (mm)	1.9	4.72

**Table 6:** Training hyperparameters.

Hyperparameter	Value
Optimizer	Adam
Learning rate	1 × 10^−3^
Loss function	Categorical crossentropy
Batch size	32
Ephocs	50

**Table 7:** Search space Keras Tuner.

Hyperparameter	Value
Learning rate	1 × 10^−5^–1 × 10^−2^
Activation function	relu, sigmoid
Number of neurons	32–512
Number of trials	10
Target (classification models)	Validation accuracy
Target (prediction models)	MAE of validation

**Table 8:** Actual force results versus measurements.

Real Data	0 g	266 g	532 g	798 g	1064 g	1330 g	1596 g	1862 g	2128 g
Measurement average	0.18	264.42	529.26	797.45	1064.58	1332.91	1599.81	1866.22	2132.53
Standard deviation	0.271	7.022	3.618	0.331	0.245	0.232	0.950	0.667	0.330

**Table 9:** Actual current results versus measurements.

Real Data	0 A	0.5 A	1 A	1.5 A	2 A	2.5 A	3 A	3.5 A	4 A	4.5 A	5 A
Measurement average	0	0.50	0.99	1.47	1.93	2.46	2.86	3.31	3.82	4.44	4.84
Standard deviation	0	0.025	0.042	0.069	0.093	0.111	0.15	0.153	0.179	0.205	0.226

**Table 10:** Results of model 1 and 2 architectures.

Architecture	Model 1 Validation Accuracy (%)	Model 1 Prediction Time (s)	Model 2 Validation Accuracy (%)	Model 2 Prediction Time (s)
LeNet-5	90.40	0.038	91.92	0.048
MobileNet	90.40	0.053	91.92	0.036
ResNet50	90.40	0.167	91.92	0.108
DenseNet201	90.40	0.538	91.92	0.337

**Table 11:** Results of model 3 and 4 architectures.

Architecture	Model 3 Validation MAE	Model 3 Prediction Time (s)	Model 4 Validation MAE	Model 4 Prediction Time (s)
LeNet-5	0.0480	0.038	0.0494	0.026
MobileNet	0.5121	0.052	0.5311	0.034
ResNet50	0.6035	0.165	1.3882	0.107
DenseNet201	0.0483	0.521	0.0491	0.337

**Table 12:** Results of model 5 and 6 architectures.

Architecture	Model 5 Validation MAE	Model 5 Prediction Time (s)	Model 6 Validation MAE	Model 6 Prediction Time (s)
LeNet-5	0.1651	0.039	0.1687	0.024
MobileNet	0.4155	0.053	0.4972	0.034
ResNet50	0.5983	0.177	0.6131	0.107
DenseNet201	0.1689	0.549	0.1680	0.330

**Table 13:** Search results for hyperparameters LeNet-5 architecture of model 1.

Test	Learning Rate	Activation Function	Number of Neurons	Validation Accuracy	Testing Accuracy
6	1.41 × 10^−5^	relu	32	93.18%	94.44%
5	4.60 × 10^−4^	sigmoid	320	89.90%	93.94%
4	1.96 × 10^−5^	relu	16	93.43%	93.43%

**Table 14:** Search results for hyperparameters LeNet-5 architecture of model 2.

Test	Learning Rate	Activation Function	Number of Neurons	Validation Accuracy	Testing Accuracy
3	1.28 × 10^−3^	relu	320	97.98%	90.91%
6	7.45 × 10^−4^	relu	160	97.47%	88.89%
10	3.76 × 10^−5^	relu	448	96.97%	88.89%

**Table 15:** Search results for hyperparameters LeNet-5 architecture of model 3.

Test	Learning Rate	Activation Function	Number of Neurons	Validation MAE	Testing MAE
2	1.27 × 10^−4^	relu	352	0.0373	0.0443
10	4.43 × 10^−5^	relu	384	0.0373	0.0456
6	2.86 × 10^−5^	relu	256	0.0366	0.0532

**Table 16:** Search results for hyperparameters LeNet-5 architecture of model 4.

Test	Learning Rate	Activation Function	Number of Neurons	Validation MAE	Testing MAE
4	5.40 × 10^−5^	relu	128	0.04328	0.04316
3	9.51 × 10^−5^	relu	352	0.04513	0.04424
2	4.08 × 10^−5^	sigmoid	128	0.04733	0.04499

**Table 17:** Search results for hyperparameters LeNet-5 architecture of model 5.

Test	Learning Rate	Activation Function	Number of Neurons	Validation MAE	Testing MAE
3	1.46 × 10^−5^	relu	288	0.0818	0.0746
2	2.67 × 10^−5^	relu	64	0.0832	0.0792
6	7.41 × 10^−4^	relu	192	0.0873	0.0844

**Table 18:** Search results for hyperparameters LeNet-5 architecture of model 6.

Test	Learning Rate	Activation Function	Number of Neurons	Validation MAE	Testing MAE
10	6.99 × 10^−4^	relu	64	0.11793	0.10248
4	1.03 × 10^−4^	relu	192	0.14070	0.08269
5	9.95 × 10^−5^	relu	192	0.14582	0.09591

**Table 19:** Cross-validation comparison models 1 and 2.

Metric	Model 1		Model 2	
	**Avg.**	**Stdev.**	**Avg.**	**Stdev.**
Accuracy	0.990	0.016	0.973	0.030
Precision	0.991	0.015	0.970	0.031
Recall	0.990	0.016	0.970	0.031
F_1_ Score	0.990	0.016	0.970	0.031

**Table 20:** Cross-validation comparison models 3, 4, 5 and 6.

Metric	Model 3		Model 4		Model 5		Model 6	
	**Avg.**	**Stdev.**	**Avg.**	**Stdev.**	**Avg.**	**Stdev.**	**Avg.**	**Stdev.**
MSE	0.0002	0.0006	0.0011	0.0014	0.00053	0.00178	0.00912	0.01041
RMSE	0.0109	0.0118	0.0255	0.0227	0.01362	0.01919	0.08413	0.04660
MAE	0.0079	0.0083	0.0186	0.0160	0.00907	0.01163	0.05644	0.03200
RAE	0.0624	0.0653	0.1454	0.1259	0.05238	0.06668	0.32538	0.17073
RSE	0.0143	0.0402	0.0615	0.0794	0.00987	0.03400	0.21143	0.32531
CC	0.9942	0.0167	0.9750	0.0330	0.99590	0.01418	0.92457	0.08726
R^2^	0.9873	0.0339	0.9428	0.0717	0.99155	0.02812	0.85745	0.1520367

**Table 21:** Comparison of models 1 and 2.

Sample ID	Target Value	Model 1	Model 2
		**Classification**	**Classification**
480	Bad	Bad (0.9999)	Bad (0.0369)
474	Good	Good (1.000)	Good (0.9628)
385	Expulsion	Expulsion (0.9999)	Expulsion (0.0505)

**Table 22:** Comparison of models 3 and 4.

Sample ID	Attribute	Target Value	Model 3	Model 4
			**Prediction**	**Prediction**
480	Pull force (N)	1410.3	1402.24	1401.82
474	Pull force (N)	2902.6	2908.43	2893.74
385	Pull force (N)	3131.7	3134.94	3126.63
480	Nugget diameter (mm)	1.90	1.8920	1.8817
474	Nugget diameter (mm)	3.59	3.5886	3.5824
385	Nugget diameter (mm)	3.47	3.4712	3.4759

**Table 23:** Comparison of models 5 and 6.

Sample ID	Attribute	Target Value	Model 5	Model 6
			**Prediction**	**Prediction**
480	Angle (°)	0	−0.0678	0.2480
474	Angle (°)	0	0.0390	−0.9022
385	Angle (°)	15	14.9465	15.8960
480	Pressure (PSI)	60	58.9396	67.4020
474	Pressure (PSI)	60	59.5769	66.9101
385	Pressure (PSI)	60	58.8801	66.1928
480	Thickness (mm)	1.250	1.2535	1.3646
474	Thickness (mm)	1.253	1.2657	1.2815
385	Thickness (mm)	1.242	1.2535	1.2576
480	Welding Time (ms)	1200	1189.48	1021.28
474	Welding Time (ms)	1200	1197.33	1199.89
385	Welding Time (ms)	600	589.64	605.423
480	Force Min (N)	98.19	99.02	83.4797
474	Force Min (N)	98.26	99.31	94.8255
385	Force Min (N)	94.93	95.49	95.2123
480	Force Max (N)	98.50	98.46	92.6841
474	Force Max (N)	98.78	99.51	101.1457
385	Force Max (N)	95.52	95.63	93.7294
480	Current Max (A)	2343.11	2319.85	3319.02
474	Current Max (A)	4251.29	4236.03	3723.13
385	Current Max (A)	4116.03	4108.54	4043.39

## Data Availability

The data presented in this study are openly available in Mendeley at doi: 10.17632/rwh8kjzdch.3 (accessed on 20 January 2025).

## Abbreviations

The following abbreviations are used in this manuscript:

A	Ampers
AISI	American Iron & Steel Institute
AQL	Acceptable Quality Level
AWS	American Welding Society
CART	Classification and Regression Tree
CC	Correlation Coefficient
CNN	Convolutional Neural Network
CSV	Comma-separated values
DP	Dual-phase
ID	Identification
IF	Interfacial Failure
ISO	International Organization for Standardization
MAE	Mean Absolute Error
MSE	Mean Squared Error
μm	Micrometers
mm	Millimeters
ms	Milliseconds
N	Newtons
nm	Nanometers
PCA	Principal Component Analysis
PF	Pull-out Failure
Q&P	Quenching and Partitioning
RAE	Relative Absolute Error
RSE	Relative Squared Error
RMSE	Root Mean Squared Error
RSW	Resistance Spot Welding
TRIP	Transformation Induced Plasticity
SPS	Samples Per Second
